# LocARNAscan: Incorporating thermodynamic stability in sequence and structure-based RNA homology search

**DOI:** 10.1186/1748-7188-8-14

**Published:** 2013-04-20

**Authors:** Sebastian Will, Michael F Siebauer, Steffen Heyne, Jan Engelhardt, Peter F Stadler, Reiche, Rolf Backofen

**Affiliations:** 1 Bioinformatics Group, Department of Computer Science, and Interdisciplinary Center for Bioinformatics, University of Leipzig, Härtelstraße 16 -18, Leipzig D-04107, Germany; 2 Bioinformatics Group, Department of Computer Science, Albert-Ludwigs-Universität Freiburg, Georges-Köhler-Allee 106, Freiburg D-79110, Germany; 3 Genetics Group, Max Planck Institute for Evolutionary Anthropology, Deutscher Platz 6, Leipzig D-04104, Germany; 4 Young Investigators Group Bioinformatics and Transcriptomics, Department Proteomics Helmholtz Centre for Environmental Research – UFZ, Permoserstraße 15, Leipzig D-04318, Germany; 5 RNomics Group, Fraunhofer Institute for Cell Therapy and Immunology, Perlickstraße 1, Leipzig D-04103, Germany; 6 Max Planck Institute for Mathematics in the Sciences, Inselstraße 22, Leipzig D-04103, Germany; 7 Center for non-coding RNA in Technology and Health, University of Copenhagen, Grønnegårdsvej 3, Frederiksberg C DK-1870, Denmark; 8 Santa Fe Institute, 1399 Hyde Park Rd., Santa Fe, NM 87501, USA

## Abstract

**Background:**

The search for distant homologs has become an import issue in genome annotation. A particular difficulty is posed by divergent homologs that have lost recognizable sequence similarity. This same problem also arises in the recognition of novel members of large classes of RNAs such as snoRNAs or microRNAs that consist of families unrelated by common descent. Current homology search tools for structured RNAs are either based entirely on sequence similarity (such as blast or hmmer) or combine sequence and secondary structure. The most prominent example of the latter class of tools is Infernal. Alternatives are descriptor-based methods. In most practical applications published to-date, however, the information contained in covariance models or manually prescribed search patterns is dominated by sequence information. Here we ask two related questions: (1) Is secondary structure alone informative for homology search and the detection of novel members of RNA classes? (2) To what extent is the thermodynamic propensity of the target sequence to fold into the correct secondary structure helpful for this task?

**Results:**

Sequence-structure alignment can be used as an alternative search strategy. In this scenario, the query consists of a base pairing probability matrix, which can be derived either from a single sequence or from a multiple alignment representing a set of known representatives. Sequence information can be optionally added to the query. The target sequence is pre-processed to obtain local base pairing probabilities. As a search engine we devised a semi-global scanning variant of LocARNA’s algorithm for sequence-structure alignment. The LocARNAscan tool is optimized for speed and low memory consumption. In benchmarking experiments on artificial data we observe that the inclusion of thermodynamic stability is helpful, albeit only in a regime of extremely low sequence information in the query. We observe, furthermore, that the sensitivity is bounded in particular by the limited accuracy of the predicted local structures of the target sequence.

**Conclusions:**

Although we demonstrate that a purely structure-based homology search is feasible in principle, it is unlikely to outperform tools such as Infernal in most application scenarios, where a substantial amount of sequence information is typically available. The LocARNAscan approach will profit, however, from high throughput methods to determine RNA secondary structure. In transcriptome-wide applications, such methods will provide accurate structure annotations on the target side.

**Availability:**

Source code of the free software LocARNAscan 1.0 and supplementary data are available at http://www.bioinf.uni-leipzig.de/Software/LocARNAscan.

## Background

Over the last decade, a series of large-scale transcriptome projects has profoundly changed our perception of the transcriptome. Reviewed e.g. in [1], pervasive transcription is widespread and plays a crucial role in controlling gene expression and genomic plasticity. Gene prediction and gene annotation of non-protein coding entities have remained non-trivial problems, nevertheless. In part, this is due to our incomplete understanding of the diversity of ncRNAs, of which novel types and subtypes keep being discovered at a rapid pace. An important confounding factor, however, is the rapid evolution of many ncRNA sequences [2-4], which intrinsically limits the applicability of homology search methods [5,6] and hence hide distant homologs.

The three-dimensional structure is important for the functionality and/or the proper processing of a large and important subgroup of ncRNAs. The most prominent representatives are ribosomal RNAs (rRNAs), transfer RNAs (tRNAs), spliceosomal RNAs (snRNAs), small nucleolar RNAs (snoRNAs), and microRNAs (miRNAs). While rRNAs and tRNAs are among the best-conserved sequences also at sequence level, other classes such as C/D box and H/ACA box snoRNAs exhibit sometimes very large substitution rates. The conservation of spatial structure implies that secondary structure, i.e., base pairing patterns, are also under stabilizing selection. In many cases, the structure evolves much slower than the sequence, see [7] for a recent detailed analysis of this phenomenon. Thus, several computational tools have been devised to utilize secondary structure alongside with sequence information for homology search. The same effect is exploited by tools such as RNAz[8], Evofold[9], or SISSIz[10] that detect selection pressure on RNA secondary structure in multiple sequence alignments.

Structural similarity is either inherited from a common ancestor or arises by convergent evolution as the result of similar selective constraints. Operationally, we distinguish *RNA families* and *RNA classes*. The members of RNA families share a sufficiently high level of sequence similarity to establish the existence of a common ancestor, which in practice translates to the possibility of representing them as structure-annotated multiple sequence alignment. The Rfam database [11] serves as a comprehensive repository for this type of data. Representatives of RNA classes share secondary structures (e.g. as a consequence of a common processing pathway in the case of microRNA precursors) or a combination of sequence and structure features (e.g. as a consequence of being incorporated into analogous ribonucleoproteins in the case of snoRNAs).

Homology search programs are geared towards detecting novel members of known RNA families, reviewed e.g. in [12]. The most commonly used tool Infernal[13] uses covariance models (CMs), i.e., the stochastic context free grammar analogue of profile hidden Markov models. Similar to search heuristics such as Erpin[14], the CMs are trained from sequence alignments that are annotated by a consensus secondary structure. When a lack of known examples precludes the construction of consensus models, tools such as RSEARCH[15] and BlastR[16] allow single structure-annotated or even unstructured RNAs as queries. A common feature of all these methods is that they heavily (or even exclusively) rely on the sequence information contained in the query model, and that they evaluate whether a piece of the target sequence *can* be folded to match a prescribed query structure. Consistency with the query structure, however, does not necessarily imply that a putative homolog is thermodynamically predisposed to actually fold into this structure. Whether the query structure is close to the target’s groundstate or whether it is an unfavourable high energy structure, therefore can provide additional information to improve specificity.

The members of *RNA classes*, on the other hand, share structural features and some sequence motifs deriving from common binding partners and functions. The term *RNA clan*[17] has been proposed for RNAs that derive from a common ancestor but have diverged far enough to either be difficult to align or have distinct functions, or both. As the distinction between clans and classes requires detailed knowledge of their evolutionary histories, we will not distinguish between clans and classes in this contributions. Examples of RNA classes are animal microRNAs (featuring a characteristic precursor hairpin and processing pattern) or the two distinct classes small nucleolar RNAs (snoRNAs) defined by the C/D and H/ACA “boxes” (short common sequence motifs) and very different characteristic secondary structures. These three paradigmatic RNA classes each comprise large numbers of families. Computational surveys [18-21], furthermore, gathered convincing evidence for large numbers of conserved RNA structures; subjecting these data sets to structure-based clustering suggested the existence of additional, so far undescribed RNA classes [22].

There are mainly two approaches for the search for novel members of RNA classes. One class consists of descriptor-based methods [23-25] or with the help of class-specific tools that combine an efficient filtering step with elaborate, often machine-learning based, evaluation procedure that ensure the required specificity [26]. In either case an in-depth knowledge of the RNA class under consideration is required. The other class does not require in-depth information on the RNA-class and its characteristic patterns of conservation. Instead it exploits information on sequential and structural similarity directly by using sequence-structure alignment. The first practical approaches for multiple structural alignment, such as RNAforester[27] and MARNA[28], depend on predicted or known secondary structures. In practice, however, these approaches are limited by the low accuracy of non-comparative structure prediction. For this reason, several derivatives of the Sankoff-based approach [29] of simultaneous alignment and folding have been introduced. In approaches such as FoldAlign[30,31], Dynalign[32], and Stemloc-AMA[33] a full energy model for RNA is implemented that is evaluated during the alignment computation. However, in its full form, these approaches suffer from a high worst case computational complexity of $O(n^{6})$ time and $O(n^{4})$ space. In contrast, PMcomp[34] and LocARNA[22] use a full-featured energy model in a precomputation step to determine a reduced representation of the structure ensemble in form of base pair probability matrices [35]. During the alignment process, base pair probabilities are used to assess the similarity of the secondary structures. Using additional computational optimizations, the complexity of LocARNA could be reduced to quartic time and quadratic memory consumption, making it currently one of the most efficient versions of the Sankoff algorithm. Several improvements and extensions of LocARNA have been discussed before: to additionally reduce LocARNA’s runtime, ExpaRNA-P[36,37] utilizes a fast structural filtering method based on local structural motifs [38,39]; REAPR[40] introduces a multiple alignment-based banding method to realign eukaryotic whole genome alignments based on RNA structure; recently, [41] introduces the very efficient LocARNA descendant SPARSE; and LocARNA-P[42] extends LocARNA by computing reliabilities, thus enabling new applications of Sankoff-style alignment. None of these approaches, however, addressed efficient scanning.

In practice, homology search relies predominantly on the sequence information in the query. Even in the CMs representing the heavily structured Rfam alignments sequence information by far outweighs the additional bit score contributed by the consensus secondary structure ( [43], Figure one point nine). Indeed, for most Rfam families, the structural information is well below the 20 bits that would be required to push the *E*-value below 1 in a small, 1M bacterial genome in a structure-based search that completely ignored sequence conservation.

CM methods ask how well the target sequence matches the query’s sequence and structure model that is derived from the input alignment and its provided consensus structure. However, they do not take into account the thermodynamic stability of the target sequence folding into the consensus structure. This could provide an additional source of information on the structural concordance of query and target.

In principle, this information is accessible in two quite different ways. Following the philosophy of Thermodynamic Matchers [25] and of the structure conservation index [44], one can interpret the consensus structure as constraint and evaluate the energy difference between constrained and unconstrained folding. The alternative is to predict structures also for the target.

A pilot study [45] provided first indications that structural alignments could be used for genome-wide homology search in a regime where sequence information is scarce. In an approach to find class-members, a model specified as base pairing probability matrix, possibly augmented with some additional sequence information, is searched against a target for which local base pairing probabilities are provided. To this end, base pairing probability matrices were computed with McCaskill’s partition function folding algorithm [35]. These were then aligned locally with LocARNA[22]. Here we describe and evaluate an optimized semi-global scanning variant of the LocARNA algorithm that can be employed in genome-wide applications.

## The LocARNAscan algorithm

LocARNA is a computationally light-weight and very efficient variant of the Sankoff algorithm [29]. It improves the CPU and memory requirements each by a quadratic factor over the original algorithm. For this purpose, LocARNA allows for matches of base pairs that occur with a given minimum probability in the structure ensembles of the single input sequences.

Here we devise a scanning variant of LocARNA, called LocARNAscan, that computes alignments of a *query* RNA with a much longer *target* sequence based on sequence and structure similarity. In our discussion, we require such alignments to be *semi-global*; in such an alignment the entire query is compared to a subsequence of the target. We achieve this by allowing free end gaps, i.e. arbitrary long deletions at both ends of the target. This semi-global scenario is designed for the common case of known motif boundaries in the query. Nevertheless, our method can be adapted to align locally with respect to both target and query as long as the locality is of biological origin. Technical locality, which was introduced in the trCYK algorithm [46] as a means of dealing with partial sequences from RNA-seq data, cannot easily be integrated, however.

We provide a generic description of the algorithm, where the target is a single sequence and, in general, the query is a multiple alignment. Furthermore, both, query and target are annotated with a base pair probability matrix, which works similarly to a weighted contact map. In general, such matrices can be computed by McCaskill’s algorithm [35]. In the common case of known query structure, we generate an appropriate base pair probability matrix by setting the probabilities of the query structure base pairs to 1 (and all others to 0). For the target, we suggest to compute local base pair probabilities by RNAplfold[45,47], which limits the span (*j* − *i*) of base pairs (*i*,*j*). Thus, we assume a maximum span *L* << *n* of target base pairs that we consider for the comparison to the query. This restriction of the base pair span serves two purposes. First, the restriction allows predicting the base pair probabilities much more efficiently. Second, RNA structure prediction methods generally tend to mispredict large base pairs. As a consequence, the accuracy is usually even increased by limiting the size of base pairs (cf. [48].)

### Notation and scoring model

Both, target *T* and query *Q* are sequences of nucleotides or, in the case of multiple alignment, alignment columns with lengths *n* and *m*, respectively. The sequences are annotated with respective substochastic matrices *P*^*T*^ ∈ [0,1]^*n*×*n*^ and *P*^*Q*^ ∈ [0,1]^*m*×*m*^. We write (*i*,*j*) ∈ *P*^*X*^ as shorthand for *P*^*X*^(*i*,*j*) ≥ *p*_*min*_(*X* ∈ {*T*,*Q*}), where *p*_*min*_ is a fixed cutoff probability. Note that for each fixed *i*, the number of *j* satisfying (*i*,*j*) ∈ *P*^*X*^ is constantly bounded, since $\underset{j}{\sum }P^{X}(i,j)\leq 1$ (cf. LocARNA [22]).

An alignment A of *T* and *Q* is a set of pairs (*i*,*j*) of indices *i* of *T* and *j* of *Q*, where all pairs (i,j),(i′,j′)∈A satisfy (*i* < *i*′ iff *j* < *j*′) and (*i* = *i*′ iff *j* = *j*′).

A *secondary structure of length n* is a set *R* of base pairs (*i*,*j*) with 1 ≤ *i* < *j* ≤ *n*, where base pairs do not cross, i.e. no two base pairs (*i*,*j*) ∈ *R* and (*i*′,*j*′) ∈ *R* satisfy *i* < *i*′ < *j* ≤ *j*′ or *i* ≤ *i*′ <*j* < *j*′.

Together with an alignment A of target and query, we are going to predict a consensus structure S. A consensus structure is a set of pairs of base pairs ((*i*,*j*),(*k*,*l*)), where the set of the respective first and second components are secondary structures of *T* and *Q*; it is *consistent with an alignment *A iff for all ((*i*,*j*),(*k*,*l*)), (i,k)∈A and (j,l)∈A.

The score of a consistent pair (A,S), which represents a *sequence-structure alignment*, is of the form

(1) $$\begin{array}{lll} \;\underset{((i,j),(k,l))\in S}{\sum } & \left[\Psi_{\left(i,j\right)}^{T}\right)+\left(\Psi_{\left(k,l\right)}^{Q}+\tau (T_{i},T_{j};Q_{k},Q_{l})\right]\; & \;\\ & +\underset{(i,k)\in A^{s}}{\sum }\sigma (T_{i},Q_{k})+\gamma N_{\text{gap}}.\; \end{array}$$

Here, $A^{s}$ denotes the set of single-stranded alignment edges A∖{(i,k),(j,l)∣((i,j),(k,l))∈S}, i.e. the alignment A without all edges that match base pairs in S. Furthermore, *γ* < 0 is the gap penalty and *N*_gap_ counts the scored gaps in A. Recall that for semi-global alignment, we allow free, non-scored, deletions at both ends of the target; thus, for the semi-global score

(2) $$\begin{array}{lll} \;N_{\text{gap}} & =\max\{i|(i,k)\in A\}\; & \;\\ & \;-\min\{i|(i,k)\in A\}+1+m-2|A|;\; \end{array}$$

the global score is of the same form of Equation (1), where $N_{\text{gap}}=n+m-2|A|$. The functions *σ*(*T*_*i*_,*Q*_*k*_) and *τ*(*T*_*i*_,*T*_*j*_;*Q*_*k*_,*Q*_*l*_) yield sequence-based similarities between elements of target and query in the unpaired and paired part of the consensus structure, respectively. We are going to discuss their instantiations later. The explicit dependence of *τ* on the aligned base pairs can be used to include contributions based on covariation or substitution. Finally, $\Psi_{a}^{T}+\Psi_{b}^{Q}$ scores the structural contribution of the consensus base pair (a,b)∈S. As in LocARNA, $\Psi_{a}^{X}(X\in \{Q,T\})$ is derived as log odd from the base pair probability *P*^*X*^(*a*), where we set Ψ^*X*^(*a*) := −*∞* if *P*^*X*^(*a*) < *p*_min_ to rule out base pairs with very low probability in (finitely scoring) consensus structures.

For simplicity, we present this score for linear (non-affine) and position independent gap cost, since the extensions of the presented method are straightforward.

Finally, we define the *subscore for i*…*j* and *k*…*l* (denoting respective subsequences of *T* and *Q*) to be of the form of Equation (1), but – unlike the total score – valid only for alignments A⊂{i,…,j}×{k,…,l}, where furthermore *N*_gap_ is defined as

j−i+1+l−k+1−2|A|,

such that the subscore penalizes deletions at both ends of the target subsequence *i*…*j*.

Finally, we define the analogous subscore *with free left end deletions* by defining *N*_gap_ as

j−min{i∣(i,k)∈A}+1+l−k+1−2|A|.

### Dynamic programming recursions

For maximizing the semi-global score of (A,S), we start by introducing dynamic programming matrices *S* and *D* in analogy to the dynamic programming matrices of LocARNA[22] and PMcomp[34]. Thus, we define the entry *S*_*i*,*j*;*k*,*l*_ as the best subscore for *i*…*j* and *k*…*l*. Furthermore, let *D*_*i*,*j*;*k*,*l*_ be the best subscore for *i*…*j* and *k*…*l* of an alignment and consensus structure S subject to the constraint that S contains ((*i*,*j*),(*k*,*l*)).

Equivalently, we redefine *S* and *D* recursively by

(3) $$\begin{array}{l} S_{i,j;k,l}=\max\left\{\begin{array}{l} S_{i+1,j;k,l}+\gamma ,\\ S_{i,j;k+1,l}+\gamma ,\\ S_{i+1,j;k+1,l}+\sigma (T_{i},Q_{k}),\\ \underset{\begin{array}{c} (i,j^{\prime })\in P^{T}\\ (k,l^{\prime })\in P^{Q} \end{array}}{\text{max}}\left(D_{i,j^{\prime };k,l^{\prime }}+S_{j^{\prime }+1,j;l^{\prime }+1,l}\right) \end{array}\right)\; \end{array}$$

(4) $$\begin{array}{lll} \;D_{i,j,k,l} & =S_{i+1,j-1,k+1,l-1}+\Psi_{\left(i,j\right)}^{T}\; & \;\\ & \;+\Psi_{\left(k,l\right)}^{Q}+\tau (T_{i},T_{j};Q_{k},Q_{l})\;\; \end{array}$$

with appropriate initializations

(5) $$S_{j+1,j;k,l}=\gamma (l-k)\;\text{and}\;S_{i,j;l+1,l}=\gamma (j-i)$$

for 1 ≤ *i* < *j* ≤ *n* and 1 ≤ *k* < *l* ≤ *m*.

Since these equations are left reducing variants of the otherwise well known recursions of PMcomp[34], one computes all entries in *D* in $O(n^{2}m^{2})$ time and O(nm) space by applying the evaluation strategy of LocARNA, which takes advantage of filtering base pairs by probability *p*_min_. However, note that the dependency on the target size *n* is still unacceptable for our purpose (of scanning genome-sized targets); therefore, we are going to improve on these complexities later.

The matrices *S* and *D* are defined to compute global subscores (i.e., without free end gaps). Since we finally need alignments with free end gaps, we have to introduce an additional DP matrix *S*^∗^. Furthermore, since we need the semi-global alignment score only for the entire target and query, it suffices to define the matrix only for scores of target and query prefixes. Thus, define $S_{j;l}^{*}$ non-recursively as the best subscore for 1…*j* and 1…*l* with free left end deletions. This is equivalent to the recursion

(6) $$S_{j;l}^{*}=\max\left\{\begin{array}{l} S_{j-1;l}^{*}+\gamma ,\\ S_{j;l-1}^{*}+\gamma ,\\ S_{j-1;l-1}^{*}+\sigma (T_{j},Q_{l}),\\ \underset{\begin{array}{c} (i^{\prime },j)\in P^{T}\\ (k^{\prime },l)\in P^{Q} \end{array}}{\text{max}}\left(S_{i^{\prime }-1,k^{\prime }-1}^{*}+D_{i^{\prime },j;k^{\prime },l}\right), \end{array}\right)$$

where the free end gaps are achieved purely by the specific initialization

(7) $$S_{j,0}^{*}=0\;S_{0,l}^{*}=\gamma l$$

for 0 ≤ *j* ≤ *n* and 0 ≤ *l* ≤ *m*.

### Efficient evaluation of the recursion equations

As mentioned before, the computational demands of the straightforward evaluation of these recursions are unacceptable for scanning. This holds even when applying the evaluation strategy of LocARNA, which never stores more than O(nm)*S* entries at each time. Although, as suggested before, we generally limit the maximum base pair span to *L*, this leaves us with prohibitive linear space dependency on the target size, since O(nm) entries of *D* have to be kept in memory before the *S*^∗^ recursion can be evaluated. Furthermore, storing the entire matrix *S*^∗^ requires O(nm) space.

**Figure 1:**
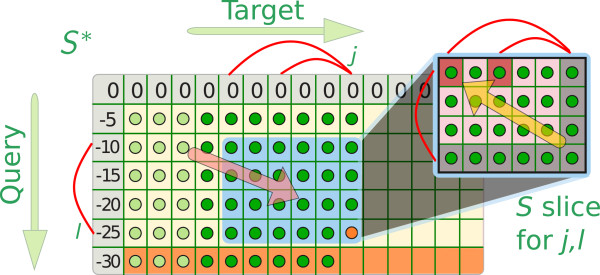
**Schematic view of the computation of *S***^***∗***^** by ****LocARNAscan****.** The dark green dots in $S_{j,l}^{*}$ are sufficient to calculate the current entry $S_{j,l}^{*}$, given the set of arcs indicated by red lines.

Therefore, we rearrange the evaluation interleaving the computation of entries in *D* and *S*^∗^. This allows us to compute all *S*^∗^ entries in one pass over the target sequence, while successively materializing required matrix entries and in turn removing “old” entries that are not accessed anymore (Algorithm 1, cf. Figure 1).

#### Algorithm 1: LocARNAscan algorithm

Computing and storing the *D* entries in lines 3–5 requires to compute and store the subset of *S* entries that allows to derive *D*(*i*,*j*,*k*,*l*) efficiently from *S*(*i* + 1,*j* − 1,*k* + 1,*l* − 1). Formulating recursion (3) recursing only to entries with the same fixed right ends *j* and *l* enables an important optimization: for each *j* and *l*, we compute a matrix slice of entries in *S* that have right ends *j* − 1 and *l* − 1 and then derive all *D*(*i*,*j*,*k*,*l*) from this matrix slice. Note that we need to compute and store at most min(*j* − 1,*L*) × (*l*−1) such entries, but potentially less depending on the actual base pairs (*i*,*j*) ∈ *P*^*T*^ and (*k*,*l*) ∈ *P*^*Q*^. After the *D* entries are derived, these *S* entries are not accessed anymore and their space can be reused.

In lines 8 and 9, we free the space of all *D* and *S*^∗^ entries that cannot be accessed by subsequent algorithm steps anymore. This is guaranteed for all entries with target left end *i* ≤ *j* − *L*, since no base pair spans more than *L* positions. Consequently, the space requirements of this algorithm are bounded by the requirement to store *Lm* entries in slices of *S*, *Lm* entries of *S*^∗^, and O(Lm) entries of *D*, i.e., O(Lm) space in total.

Allocating and freeing entries can be implemented in constant time by using rotating matrices, commonly implemented by addressing based on target indices modulo *L*. Since *D* is a sparse matrix, it is conveniently implemented based on a hash. Thus, the time complexity of this algorithm is bounded by computing all matrix entries, i.e. *nm* entries of *S*^∗^, $O(\text{nm}L^{2})$ entries of *S*, and O(nm) entries of *D*. Note that each entry is computed in constant time; for *S* and *S*^∗^ this holds due to considering only a sparse subset of base pairs. Thus, we derive the total time complexity $O(\text{nm}L^{2})$.

### Sequence score contributions

The sequence contribution of our score is defined via the two similarity functions *σ* and *τ*. Note that LocARNAscan was designed to work even without sequence information. In this special case, we set both functions to constantly return 0.

In general, LocARNAscan accepts information about the query sequence in the form of a multiple alignment. Replacing the Ribosum-like [15] definition of *σ* and *τ* in LocARNA, we suggest to utilize log-odd scores based on nucleotide (and nucleotide pair) frequencies in this multiple alignment. Whereas the original LocARNA score is tailored for comparing single sequences or constructing multiple alignments (there used in a sum-of-pairs score), log-odd based scores, which are similarly utilized by Infernal[13], are more appropriate for scanning applications.

Given two column vectors *q* and *q*′ of nucleotides and gaps from the query multiple alignment and given two nucleotides *t* and *t*′ from the target, *f*_*q*_(*t*) denotes the frequency of *t* in *q*; $f_{q,q^{\prime }}(t,t^{\prime })$, the frequency of pairs *t* and *t*′ in the corresponding rows of *q* and *q*′. Let *b*_*t*_ be the background frequency of nucleotide *t*; $b_{tt^{\prime }}$, of the nucleotide pair *t*, *t*′ in canonical base pairs. The similarity in the single stranded case is then defined by

(8) $$\sigma (t,q)=\log\frac{f_{q}(t)}{b_{t}};$$

the similarity in the base paired case, by

(9) $$\tau (t,t^{\prime },q,q^{\prime })=\log\frac{f_{q,q^{\prime }}(t,t^{\prime })}{b_{tt^{\prime }}}.$$

For simplicity, we assume uniform distribution of single nucleotides and nucleotides in base pairs. That is we use background frequencies *b*_*t*_ = 1/4 and $b_{tt^{\prime }}=1/6$; the latter reflects that we consider all six canonical base pairs.

In our implementation, for fast evaluation of the similarity functions, we compute the profiles, consisting of $f_{Q_{k}}$ and $f_{Q_{k},Q_{l}}$ for all query position *k* < *l*, prior to the actual scanning. Furthermore, to handle small query alignments and smoothen the scoring functions, we add pseudocounts depending on the query’s mononucleotide background frequencies.

### Reporting optimal and suboptimal occurrences of the query

An occurrence of *Q* in *T* is a subsequence *T*[*i*]…*T*[*j*] of *T*; its score is the best subscore for *i*…*j* and 1…*m*. The optimal occurrence of *Q* in *T* is determined during the run of Algorithm 1 by recording the *j* with the best score *S*^∗^(*j*,*m*). By definition of *S*^∗^, *j* is the right end of the target subsequence that optimally aligns with the query, in the case that an arbitrarily long left end of the target can be deleted for free. Albeit finding the left end requires extra work (see next subsection), the right end suffices to specify the occurrence.

For reporting suboptimal occurrences, one can record all scores *s*_*j*_ := *S*^∗^(*j*,*m*) during the run of the algorithm. However, reporting all occurrences down to a certain score threshold in this vector is unsatisfactory, since good scores are usually flanked by only slightly worse scores that refer to the same occurrence with slightly altered alignment. For this reason, we report only local maxima of *s*_*j*_, i.e. *j*, where *s*_*j*_ ≥ max(*s*_*j*−1_,*s*_*j*+1_). Furthermore, we do not report a local maximum *j*′ if it is too close to a reported local maximum *j* with a better or equal score. Setting the distance threshold to one query length (*m*), we avoid reporting occurrences with substantial overlap. More formally, for *j* and *j*′ where |*j*−*j*′| ≤ *m*, we say that *j* *dominates* *j*′, iff $s_{j}>s_{j}^{\prime }$ or ( $s_{j}=s_{j}^{\prime }$ and *j* < *j*′). We prune all dominated local maxima and report only the non-dominated ones.

Limiting the memory consumption independent of the target length is crucial for scanning. In addition, random access to data growing linearly with the target length has to be avoided. Consequently, we devise an online pruning algorithm with space requirements linearly bounded by the query length. While scanning the target by algorithm 1, we maintain a list of local maxima *j*′ and remember their corresponding scores $s_{j^{\prime }}$. We maintain three invariants: 1) list entries are increasing, 2) the distance of successive list entries is smaller than or equal to *m*, and 3) scores of list entries are strictly increasing.

When identifying a new local maximum *j* (after computing *s*_*j* + 1_ = *S*^∗^(*j* + 1,*m*)), we try to resolve as many domination relations as possible. If *j*−*m* is larger than the last list entry, entries of the list are independent of the new local maximum and all further local maxima. Thus, the list is resolved by iteratively reporting the last list entry and removing all dominated entries. All non-dominated entries are reported and the list is deleted. Finally, unless *j* is dominated by the last *j*′ in the list, we push *j* to the list. Note that here we push to the empty list or the score *s*_*j*_ is higher than the score of the last list entry. After scanning the entire target, the list is resolved again.

The correctness follows from preserving the invariants in all cases. It remains to show that the list length is linearly bounded by the query length. This is due to the discretization of our score (we round all score contributions to limited precision); being an additive score, the score is linearly bounded by the query length; consequently, lists satisfying the third invariant have linearly bounded length. Note that in the average case, lists are even much shorter than this theoretic worst case bound suggests.

To handle large targets without excessive use of main memory, we implement two reporting strategies. Either we output all reported occurrences immediately or we limit the number of reported occurrences a priori to some *K*. To output only *K* best occurrences, we store them in a priority queue, sorted such that the occurrence with minimum score is on top and thus can be removed, whenever the limit is exceeded. Finally, the contents of the queue are output. While outputting the occurrences, we keep a histogram of the locally maximal scores for later use in determining the significance of an occurrence from the empirical score distribution. The entire strategy is generally similar to GotohScan[49] and extended here by the online pruning procedure.

**Figure 2:**
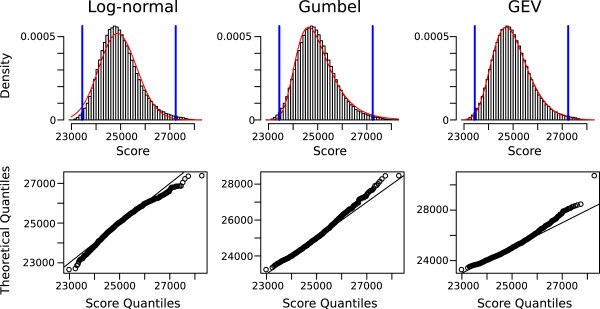
**Fitting of several commonly used probability distributions to the histogram of ****LocARNAscan****scores.** Scores correspond to LocARNAscan alignments using the profile of the RFAM family RF00504 (glycine riboswitch) as input query. The first row shows the fitting of log-normal, gumbel, and generalized extreme value (gev) distributions (red curves) to the alignment scores shown as histogram. The shown scores have been shifted to positive values. In the lower panel, we compare the distributions by Q-Q plots. These plots, which plot the quantiles of the observed scores vs. expected quantiles from the theoretical distributions, visualize in how far two distributions differ in location, scale and skew from each other. All tested known probability distributions (including normal and gamma distribution; data not shown) do not represent the LocARNAscan alignment score distribution well; visible in the Q-Q-plot, since none of the Q-Q plots follows a straight line.

To determine the significance of an occurrence, we compared several theoretical distributions to the empirical score distribution, but none approximated the alignment scores well (Figure 2). Hence, we followed the approach presented in [49]: for the identification of extreme alignment scores, it suffices to approximate the right tail of the distribution; the tail can be fitted well by an affine function. This strategy enables to return only *significant* occurrences with e-value up to a given threshold.

### Traceback and Bound on Alignment Length

Obtaining the actual alignments corresponding to occurrences with right end *j* and recovering the left end of the occurrence requires a traceback procedure through the dynamic programming matrices. This poses the problem that space limitations forbid storing the entire matrices, such that these matrices are not available after running Algorithm 1.

Therefore, given *j*, we determine and recompute the relevant part of matrix *S*^∗^ and the corresponding *D* entries. Efficiency demands to keep the recomputed relevant part of *S*^∗^ as small as possible. Given the score *s*_*j*_, we derive a lower bound *i* for relevant left ends *i*′ of the occurrence. Consequently, since the occurrence score cannot depend on entries with lower target left ends *i*′ < *i*, one initializes all *S*^∗^(*i* − 1,*k*′) with −*∞* (1 ≤ *k*′ ≤ *m*), all *S*^∗^(*j*′,0) with 0 (*i*−1 ≤ *j*′ ≤ *j*), and recomputes all entries *S*^∗^(*j*′,*k*′), where *i* ≤ *j*′ ≤ *j* and 1 ≤ *k*′ ≤ *m*, according to Eq. (6).

Finally, transferring a strategy known from LocARNA, we free *S* entries during this first recomputation, such that we recompute the *S* entries “on the trace” a second time, when performing the actual traceback. In this way, the traceback does not require more space than the score computation.

Analogously to [45], we bound the difference between the length of query and occurrence by Δ, where we choose Δ := (*s*_*j*_−*m* max(*σ*_max_,*τ*_max_/2))/*γ* such that

$$s_{j}\leq m\max(\sigma_{\max},\tau_{\max}/2)+\Delta \gamma ,$$

and *σ*_max_ and *τ*_max_ denote the respective maximum single base match and maximum base pair match contributions between query and target; notably, the latter similarity includes sequence contributions due to *τ* and structure contributions due to Ψ^*T*^ and Ψ^*Q*^.

Whereas [45] suggests to limit the “history” during the search phase by *m* + Δ, we limit this more strongly to *L* at the price of recomputation. Making the common case fast, this strategy is generally advantageous; it reduces load in the scanning phase, while only very few entries close to high scoring occurrences have to be recomputed, causing largely negligible extra cost. Similarly, one bounds the number of required entries of *D*, since the benefit of matching two base pairs has to justify a potential length difference of the enclosed subsequences.

## LocARNAscan recognizes thermodynamic stability of occurrences

To study the specific behavior of LocARNAscan, we compared the performance of LocARNAscan and Infernal on a designed target containing a series of thermodynamically stable and (presumably) unstable occurrences. This allowed us to measure the difference in sensitivity to both classes given different training information.

First, we designed two sets of 1000 RNA decoys each. In the first set, which we call *stabilized class*, the RNA decoys were designed, applying inverse folding, to fold into tRNA structures with high thermodynamic stability. In the second set, called *non-stabilized class*, the decoys were generated with the potential to fold into tRNA structures forming canonical base pairs, but their stability is purely by chance. By design, the decoys are not related on the sequence level to each other or to known RNAs but share approximately the same mononucleotide frequencies. However, each decoy has the length and structure of a different randomly selected sequence from the Rfam tRNA family. From these decoys, we generated one *pseudogenome* consisting of an RNA decoy every 200 bases padded with random nucleotides from the same mononucleotide distribution.

For each class, we selected 100 samples at random to use them as training sets. Based on the Rfam seed alignment, we obtained a multiple alignment of the 100 samples annotated with a consensus structure derived from the Rfam consensus structure of the entire family. From both multiple alignments, we generated covariance models for Infernal. From the model for the non-stabilized class, we striped off all sequence information, replacing it by a background model based on the mononucleotide frequencies of the pseudogenome. This procedure resulted in a *stripped model* and a *stabilized model*. For LocARNAscan, we generated two different queries. The first query consists of a tRNA of median length, where the sequence was replaced by a string of Ns and *P*^*Q*^ was generated from the Rfam structure of this tRNA; the second, of the “stabilized” multiple alignment and a matrix *P*^*Q*^ that was generated from its Rfam-derived consensus structure. By design, the first query contained at most the information of the stripped model, whereas the second query and the stabilized model contain exactly the same information.

We emphasize that both, the stripped CM and the all-N query sequence represent intentionally extreme cases. Since stable RNAs favor CG base pairs over AU and GU base pairs and conversely, A and U in single stranded regions over C and G, one could usually utilize such knowledge, even in the absence of sequence information. Without doubts, Infernal would profit from this knowledge; building corresponding models is even supported via cmbuild’s option eset 0 in Infernal 1.1. As well, LocARNAscan could be similarly extended to mirror Infernal’s behavior. Nevertheless, we intentionally study the extreme scenario to isolate the effect of incorporating thermodynamic stability.

Finally, we performed four scans of the pseudogenome. We run LocARNAscan with both designed queries and Infernal with the stripped model and the stabilized model. While the former model allowed Infernal to make full use of its training machinery, the latter tested Infernal’s performance without sequence information. Per query, Infernal scanned the genome in 9 minutes (on Intel Q9400 2.66 Ghz), whereas LocARNAscan took only 3:10 minutes. Using its HMM filter, Infernal improves to roughly 6 minutes. LocARNAscan additionally requires to precompute base pair probabilities by folding the genome once (for potentially many queries); RNAplfold performed this in 5 minutes.

**Figure 3:**
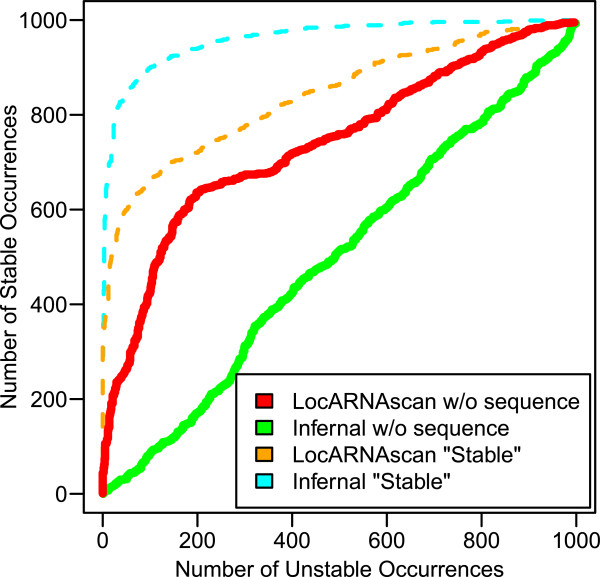
**Classification of thermodynamically stabilized *vs.* non-stabilized occurrences.**

In Figure 3, we compare the separation of the two decoy classes by the different runs by their classification behavior. For each run, we plot the number of occurrences that coincide with stabilized decoys vs. the number with non-stabilized decoys at the same score threshold. The curves show that Infernal without sequence information completely fails to distinguish the two classes; in contrast, samples of stabilized decoys contain enough sequence information to allow Infernal to classify almost perfectly. Trained without sequence information, LocARNAscan is superior to Infernal in sensing the stability of the decoys. Given the multiple alignment, LocARNAscan gains classification strength, but does not match the excellent performance of Infernal with sequence information.

## Discussion and Conclusions

Genome-wide search for homologs of structured RNAs can benefit from the additional information encoded in their base pairing patterns. Nevertheless, currently available tools predominantly utilize the sequence information contained in query and target. The structural contributions are incorporated at the level of consistency between target sequence and query structure. Here we asked whether it can be worthwhile to include direct structural information, and thus implicitly evidence for the stability of secondary structure, also on the side of the target.

This is feasible in practice based on “scanning versions” of RNA secondary structure prediction tools that compute e.g. probabilities for local base pairs with a limited span in the target. Homology search on such a structure-annotated target is naturally performed as a local or semi-global sequence-structure alignment. With LocARNAscan we devised an efficient implementation of a semi-global variant of the Sankoff algorithm. For applications of genome-wide searches, we developed several algorithmic improvements relative to the global LocARNA approach. Rethinking the trade-offs between storing and recomputing intermediate data, LocARNAscanrequires only O(Lm) memory, dependent only on the query size *m* and the span *L* of the precomputed base pairs in the target, but independent of the size *n* of the target itself, which is linearly read from disc and does not need to be stored in its entirety.

LocARNAscan’s CPU requirements of $O(\text{nm}L^{2})$ make genome-wide scanning feasible. In our experiment, LocARNAscan including the precomputation of base pair probabilities by RNAplfold, is about as fast as Infernal; this does not even change much when Infernal is allowed to use HMM filtering, since the small contribution of sequence information in our setting limits the effect of filtering. Calibration of the Infernal model, which is usually required, would have increased its total time requirements dramatically. The actual scan by LocARNAscan and the RNAplfold precomputation took almost the same time. We point out that the theoretical worst case complexities $O(\text{nm}L^{2})$ of LocARNAscan, $O(nL^{2})$ of RNAfold, and $O(nm^{2})$ of Infernal would suggest a different ordering of run times. The observed run times are a consequence of very different constant overheads. While LocARNAscan’s *L*^2^ factor is strongly reduced by the filtering of base pairs by their probability, e.g. RNAplfold’s overhead is much larger due to the complex energy model and Infernal’s run time actually depends on the number of CM states, which is a multiple of the query length.

Not surprisingly, little can be gained by the extra expense of the sequence-structure alignment as long as sufficient sequence information is contained in the query. We therefore concentrated here on the regime in which our current homology search tools are effectively blind, i.e., cases in which sequence conservation is completely absent. We find that in such an extreme setting LocARNAscan retains the ability to distinguish between thermodynamically stable RNA elements and decoys that admit the same base pairing patterns albeit far away from their groundstate. In contrast, Infernal is blind to this difference.

Of course, this is an extreme regime that is of interest in rare applications since most RNA families also exhibit an appreciable level of sequence conservation. A further practical limitation is the accuracy of the predicted base pairs of the target structure. Several approaches for the genome-wide measurements of secondary structure (see [50] and the references therein), however, promise to at least alleviate this issue in the near future.

## Methods

### Design of decoy RNAs

Each decoy (in both the stabilized and the non-stabilized class) is generated from a randomly selected entry in the Rfam 11.0 seed alignment of the tRNAs. First, we remove all gap columns and the corresponding symbols in the dot-bracket consensus structure string. If this deletes only one parenthesis of a parentheses pair, we replace the other one by a dot. In this way, we generate the ungapped tRNA sequence *S* and the corresponding projection of Rfam’s tRNA family consensus structure. Then, we fold the sequence constrained with the consensus structure projection (using RNAfold). This results in a specific structure *R* for the selected tRNA. For the stabilized class, we apply inverse folding into structure *R* optimizing the probability of *R* in the ensemble of the designed sequence *S*; for the inverse folding we apply RNAinverse in partition function mode. In addition, we configure RNAinverse to stop at a cutoff corresponding to probability *p* = 0.75. This generally results in sequences, where *R* has only slightly higher probability.

The tool RNAinverse does not directly allow to stop the optimization at a probability *p*. However, RNAinverse accepts a minimal energy difference between the energy *E*(*R*|*S*) of *R* for *S* and the ensemble free energy $F(S)=-\mathrm{RT}/\ln(\underset{\hat{R}}{\sum }E(\hat{R}|S))$ of *S*. We utilize this feature after inferring the *p*-equivalent difference by

(10) ΔE=−RT/ln(p),

where ℜ denotes the gas constant and *T* the temperature.

We generate the non-stabilized decoys based on the mononucleotide frequencies of the stabilized ones. For generating a decoy in the non-stabilized class, given a structure *R* and mononucleotide frequencies *p*_*X*_(*X* ∈ {*A*,*C*,*G*,*U*}), we draw the unpaired bases and bases at left base pair ends randomly from this distribution. The right ends of base pairs are set complementary to the left end bases (disallowing GU pairs).

### Stripping off sequence information of a covariance Model

Similar to profile hidden Markov models (HMMs), Infernal’s covariance models (CMs) are generative models that describe a probability distribution over sequences. Designed for RNAs, the CMs contain information about the RNA secondary structure, such that they can distinguish between unpaired bases and base pairs. Thus, like profile HMMs, the CMs contain nucleotide emission probabilities at different match states, but additionally contain nucleotide pair emission probabilities at special base pair match states. Technically, CMs contain log odd bit scores of the emissions calculated from the emission probabilities and, in uncalibrated CMs, a simple uniform null model. The key to entirely remove sequence information from a CM is thus to replace all emission scores of nucleotides and nucleotide pairs according to a background model.

Given mononucleotide frequencies *p*_*X*_(*X* ∈ {*A*,*C*,*G*,*U*}), we replace all emission scores for nucleotide *X* by $\log_{2}(\frac{p_{X}}{1/4})$. For base pairs, we take into account a low probability *p*_nc_ of emitting a non-canonical nucleotide pair (*p*_nc_ = 0.001). The scores for nucleotide pairs (*X*,*Y*) ∈ {*A*,*C*,*G*,*U*}^2^ are then replaced by $\log_{2}(\frac{(1-p_{\text{nc}})p_{X}p_{Y}/6}{1/16})$ for canonical pairs *XY* ∈ {*AU*,*UA*,*CG*,*GC*,*GU*,*UG*} or $\log_{2}(\frac{p_{\text{nc}}p_{X}p_{Y}/6}{1/16})$ for non-canonical pairs.

### Running LocARNAscan and Infernal

First, we performed two scans of the pseudogenome by LocARNAscan 1.0 ; the tool is available as free software at http://www.bioinf.uni-leipzig.de/Software/LocARNAscan. In extension of the presented score of Equation (1), LocARNAscan 1.0 supports affine gap cost and weighs the structure against sequence similarity. We set the weighting factor to 2.0 (option struct-weight=200) and the weighting of the sequence contribution *τ*(*T*_*i*_,*T*_*j*_;*Q*_*k*_,*Q*_*l*_) in the structure score component to 4.0 (option tau=400). Furthermore, we set the insertion/deletion score to -1.0 and gap opening cost to -5.0 (options indel=-100 and indel-opening=-500). Finally, we activate the introduced log odd scoring of LocARNAscan (option logoddscores on). Base pairs in the pseudogenome are predicted with RNAplfold[47] forbidding lonely base pairs and setting a folding window size of 200nt and a maximal base pair span of 100nt. From the RNAplfold prediction, we removed all base pairs with probability less than 0.5.

Second, we performed two corresponding scans by Infernal 1.0.2. We built Infernal models by cmbuild with default parameters. Then, we scan the pseudogenome with cmsearch. To avoid missing decoys we turned off HMM filtering (option fil-no-hmm). Furthermore, to produce comparable results, we scan only the forward strand (cmsearch option top-only) and run Infernal in *glocal* mode (option -g); the latter turns on LocARNAscan-like semi-global behavior.

For both, LocARNAscan and Infernal, we determined findings of stabilized and non-stabilized decoys and the corresponding occurrence score. There, we considered an occurrence, if it overlaps a decoy by at least 10% nucleotides. In the case of multiple occurrences overlapping the same decoy, we selected the best prediction as score for this decoy.

## Competing interests

The authors declare that they have no competing interests.

## Authors’ contributions

RB, KR, PFS, and SW designed the algorithm and study; MS and SW implemented LocARNAscan; JE, SH, MS, and SW evaluated its performance; finally, all authors contributed to writing the manuscript. All authors read and approved the final manuscript.

## Authors’ information

Sebastian Will and Michael F Siebauer are joint first author.
